# CircFOXO3 upregulation mediates the radioresistance of glioblastoma by affecting cellular metabolome

**DOI:** 10.3389/fphar.2024.1479480

**Published:** 2024-10-10

**Authors:** Hao Xu, Jin Xing, Lilin Cheng, Zhihan Wang, Liang Zhao, Li Ren, Shuai Zhang

**Affiliations:** ^1^ Department of Neurosurgery, Shanghai Pudong Hospital, Fudan University Pudong Medical Center, Shanghai, China; ^2^ Department of Neurosurgery, Changhai Hospital, Naval Medical University, Shanghai, China

**Keywords:** glioblastoma, circFoxo3, metabolomics, radiotherapy, radioresistance

## Abstract

**Introduction:**

Radioresistance remains a significant challenge in the treatment of glioblastoma multiforme (GBM), the most prevalent and lethal brain cancer in adults. Metabolic alterations are known to contribute to radioresistance by activating antioxidant responses and promoting DNA repair. However, the role of circular RNAs in this process, particularly circFOXO3, is not well understood.

**Methods:**

In this study, we investigated the expression of circFOXO3 in glioma cells exposed to radiation and in recurrent GBM tissues. We performed knockdown and overexpression experiments *in vitro* and *in vivo* to assess the effects of circFOXO3 on radiosensitivity. Metabolomic profiling was conducted to explore the metabolic changes associated with circFOXO3 overexpression following irradiation.

**Results:**

Our results showed significant upregulation of circFOXO3 in glioma cells upon radiation exposure and in recurrent GBM tissues. Knockdown of circFOXO3 increased radiosensitivity both in vitro and in vivo, whereas overexpression of circFOXO3 attenuated radiosensitivity. Metabolomic analysis revealed substantial alterations in lipid and organic compound profiles between circFOXO3-overexpressing and control groups. Additionally, circFOXO3 suppression increased proapoptotic protein levels (Caspase 7 and Bax) and decreased anti-apoptotic protein Bcl-2 levels following radiotherapy.

**Discussion:**

These findings demonstrate the pivotal role of circFOXO3 in promoting tumor radioresistance through metabolic modulation, suggesting that circFOXO3 could serve as a potential diagnostic and therapeutic target for GBM.

## 1 Introduction

Glioblastoma multiforme (GBM) is a common malignant intracranial tumor with high morbidity and mortality rates. The current standard treatment for GBM involves maximal surgical resection with the goal of minimizing neurological complications, followed by radiotherapy and chemotherapy (Ostrom et al., 2022; van den Bent et al., 2023). Despite comprehensive therapeutic approaches, overall survival remains limited to only 14.6 months (Ostrom et al., 2014). Among all adjuvant treatments, radiotherapy is the primary therapeutic modality for GBM patients. However, many tumors relapse after treatment, potentially due to inherent or acquired radioresistance in tumor cells (Liu et al., 2023). Therefore, elucidating the mechanisms underlying GBM radioresistance and identifying novel molecular targets that can enhance the response to current therapeutic interventions are crucial.

A recent study highlighted the pivotal role of metabolic alterations in promoting radioresistance across various cancers (Kang et al., 2023). Notably, DNA damage repair and reactive oxygen species (ROS)-related pathways are significantly influenced by radiation exposure (Liao et al., 2023). Metabolic by‐products, such as aldehydes and alkylating agents, can cause diverse types of DNA damage, and the function and recruitment of DNA repair-related genes are also regulated by metabolic reactions. Furthermore, alterations in the metabolite composition within the tumor microenvironment can facilitate tumor growth after radiotherapy (Sedlackova et al., 2023; Gupta et al., 2020). Therefore, combining radiotherapy with targeted metabolic interventions is a promising approach for increasing treatment efficacy.

Forkhead box O3 (FOXO3), a member of the forkhead transcription factor family, contributed extensively to gene regulation by binding to and activation of enhancer regions in cancer cells (Rodriguez-Colman et al., 2024). CircFOXO3 (has_circ_0006404), which is derived from the FOXO3 gene, has been reported to be dysregulated in multiple tumors (Lu, 2017; Zhang et al., 2018), and targets its parental gene FOXO3 by translational or post-translational modification (Rodriguez-Colman et al., 2024; Su et al., 2021). However, the role of circFOXO3 in GBM radiosensitivity and related metabolism remains unclear. In this study, we performed expression analysis using fresh samples collected from recurrent GBM patients and irradiated glioma cells. CircFOXO3 levels were notably greater in both recurrent samples and irradiated cells than in normal controls. Loss-of-function analyses were conducted using a panel of glioma cell lines to investigate the effects of circFOXO3 knockdown (KD)/overexpression (OE) on GBM proliferation and DNA damage repair processes. Additionally, metabolomics analysis revealed significant changes in metabolite profiles between the circFOXO3 and control groups after irradiation, including alterations in glutathione, 6-hydroxyhexanoate, and chlorambucil levels. In addition, several apoptotic proteins were found to be altered by circFOXO3 following radiotherapy. Thus, this study provides insights into the role of circFOXO3 in GBM radioresistance and provides an initial exploration of associated metabolic changes.

## 2 Materials and methods

### 2.1 Patients and sample collection

The study was approved by the Ethics Committee of Shanghai Pudong Hospital, Fudan University Pudong Medical Center. Twenty primary tumor and twenty recurrent tumor samples were collected, and these fresh samples were used for quantitative real-time polymerase chain reaction (qRT-PCR). The primary and recurrent samples were obtained from different patients, all of whom underwent surgical resection at their initial visit and subsequently received postoperative chemoradiotherapy.

### 2.2 Irradiation

The cells or xenograft tumor models were placed in a vertical position and subjected to local irradiation to the head via a VARIAN linear accelerator with a total dose of 6 Gy, as it strikes a balance between inducing significant stress responses in glioma cells without causing excessive cell death, making it an optimal dose for studying radioresistance mechanisms.

### 2.3 Single-cell gel electrophoresis

The single-cell gel electrophoresis assay was carried out with a comet assay kit (Trevigen, United States) according to the manufacturer’s instructions. Briefly, single-cell suspensions were washed with PBS and mixed with low-melting agarose (1:10). Cell lysis was induced by incubating the mixture at 4°C for 3 h, and the treated cells were then electrophoresed for 20 min. Subsequently, the DNA was visualized by staining with 5 µg/mL Goldview (SBS Genetech, Co., Ltd.) after fixation. Digital fluorescence images were obtained.

### 2.4 γ-H2AX immunofluorescence analysis

Immunofluorescence was performed with an anti-γ-H2AX antibody (Cell Signaling Technology, United States). The nuclei were counterstained with Hoechst 33342 (Beyotime Biotechnology, Shanghai, China). The number of γ-H2AX foci was counted in at least 50 cells per condition under a laser scanning confocal microscope (LSM 700, Zeiss, Oberkochen, Germany).

### 2.5 Xenograft tumor assay

Four-week-old athymic nude mice were used for the xenograft tumor assay. All the mice underwent the same stereotactic injection procedure and were subjected to one of three different treatment conditions: 1) U87-MG-negative control (NC) without irradiation; 2) U87-MG-circFOXO3 KD without irradiation; and 3) U87-MG-circFOXO3 KD under 6 Gy irradiation. The indicated cells (1 × 10^6^) were implanted into the corpus striatum of anesthetized athymic nude mice using a small animal stereotactic frame (David Kopf Instruments). Tumor volume was calculated as (length × width (van den Bent et al., 2023))/2 using Function Analysis software (General Electric). The animal study was approved by the Institutional Animal Care and Use Committee of Shanghai Pudong Hospital, Fudan University Pudong Medical Center.

### 2.6 Untargeted metabolomics analysis

For untargeted metabolomics, T98G-circFOXO3-OE and NC cells (n = 6 per group) were subjected to liquid chromatography-mass spectrometry (LC-MS) analysis. The samples were prepared and analyzed using a quadrupole time-of-flight mass spectrometer (Sciex TripleTOF 6600) coupled with hydrophilic interaction chromatography. The data were processed using XCMS software, with a VIP value > 1 and p< 0.05 considered statistically significant. Metabolites were mapped to pathways using the Kyoto Encyclopedia of Genes and Genomes (KEGG) database. Further hierarchical clustering was performed using Cluster 3.0 and Java Treeview software.

More detailed steps are provided in the Supplementary Material.

### 2.7 Statistical analysis

The data are presented as the means ± standard errors of the means of at least three independent experiments. For comparisons between two groups, an unpaired t-test was used, and for comparisons among multiple groups, one-way analysis of variance followed by Tukey’s *post hoc* test was conducted. All the statistical analyses were performed via SPSS for Windows v.17.0 (SPSS, Chicago, IL). All the results were considered significant at a two-sided P-value < 0.05.

Details on the methods used for cell culture, qRT-PCR, Western blot analysis, lentiviral vector-mediated gene alteration, and bioinformatic analysis can be found in the Supplementary Material.

## 3 Results

### 3.1 CircFOXO3 is highly expressed in glioma cells in response to radiation

**FIGURE 1:**
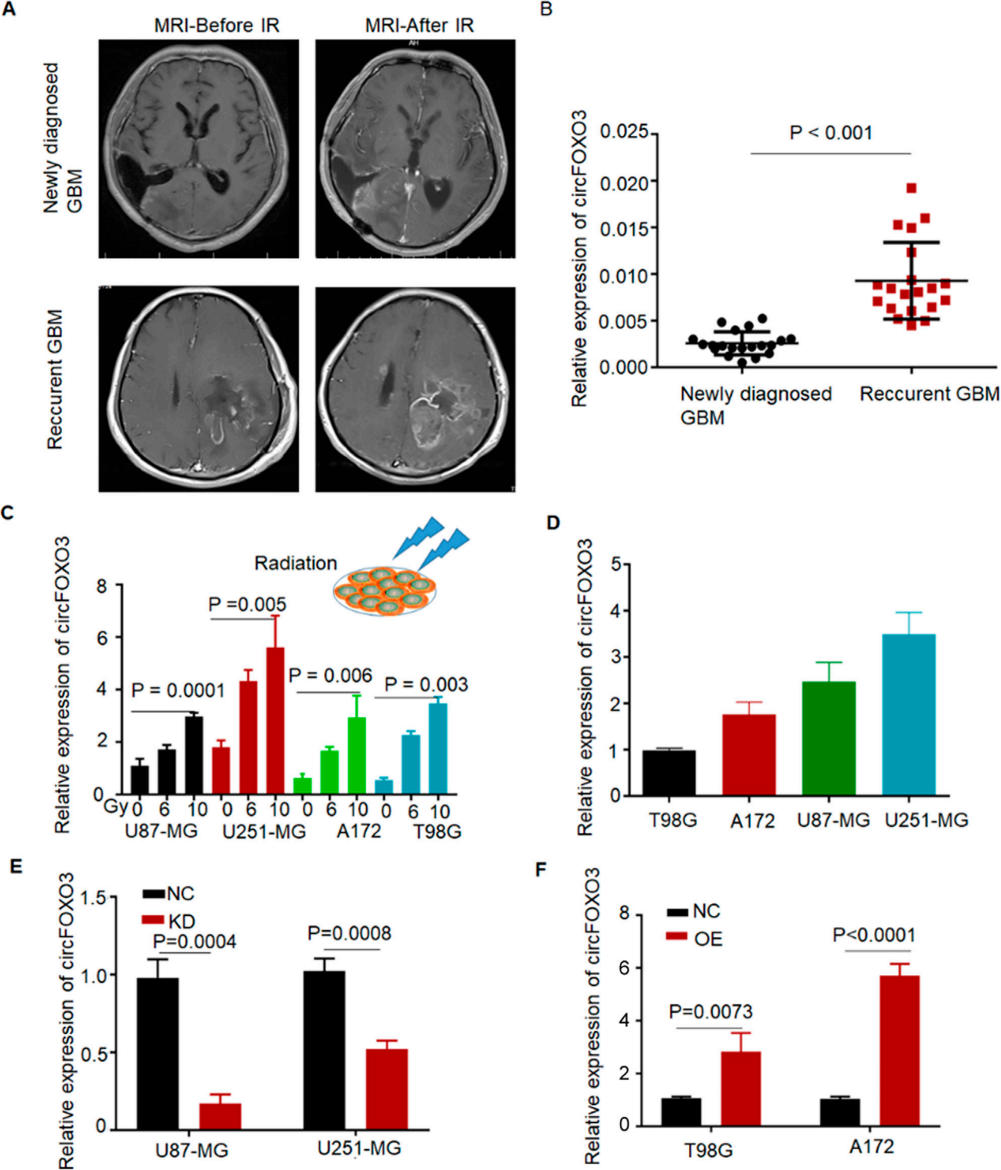
CircFOXO3 is significantly upregulated in recurrent GBM and tumor cells after irradiation. **(A)** Enhanced brain MRI of GBM tissues with or without irradiation. Patient 1had Newly diagnosed GBM; Patient 2 had recurrent GBM. **(B)** CircFOXO3 expression in newly diagnosed GBM and recurrent GBM. **(C)** Relative expression of circFOXO3 in glioma cells after 0, 6 and 10 Gy irradiation. **(D)** Relative expression of circFOXO3 in glioma cells. **(E)** CircFOXO3 KD in U87-MG and U251-MG cells by lentivirus transfection; inhibition of circFOXO3 is shown. **(F)** CircFOXO3 OE in T98G and A172 cells by lentivirus transfection. The data are presented as the mean ± SD.

A part of patients with recurrent GBM exhibited tumor progression on magnetic resonance imaging (MRI) within 30 days after radiotherapy (Figure 1A). To quantify the RNA levels of circFOXO3, qRT‒PCR analysis was performed on recurrent and primary GBM tissues. The results revealed that circFOXO3 expression was significantly higher in recurrent GBM patients than in newly diagnosed patients (Figure 1B).

The expression levels of circFOXO3 in A172, U87-MG, U251-MG, and T98G cells were also quantified after exposure to 0, 6, and 10 Gy irradiation. Compared with that in untreated cells, a significant dose-dependent increase in circFOXO3 was observed in irradiated cells compared to untreated cells, indicating that circFOXO3 expression changes in glioma cells upon radiation exposure (Figure 1C). These findings strongly suggest a critical association between circFOXO3 and radioresistance in glioma.

### 3.2 Inhibition of circFOXO3 increases the radiosensitivity of GBM *in vitro*

To explore the role of circFOXO3 in GBM radiotherapy, we constructed stable circFOXO3 KD and OE cell lines via lentiviral vectors. As shown in Figure 1D, T98G and A172 cells presented decreased circFOXO3 expression, whereas U87-MG and U251-MG cells presented elevated circFOXO3 levels. Efficient inhibition of circFOXO3 expression was confirmed in circFOXO3-KD cells compared with the NC cells (Figure 1E).

**FIGURE 2:**
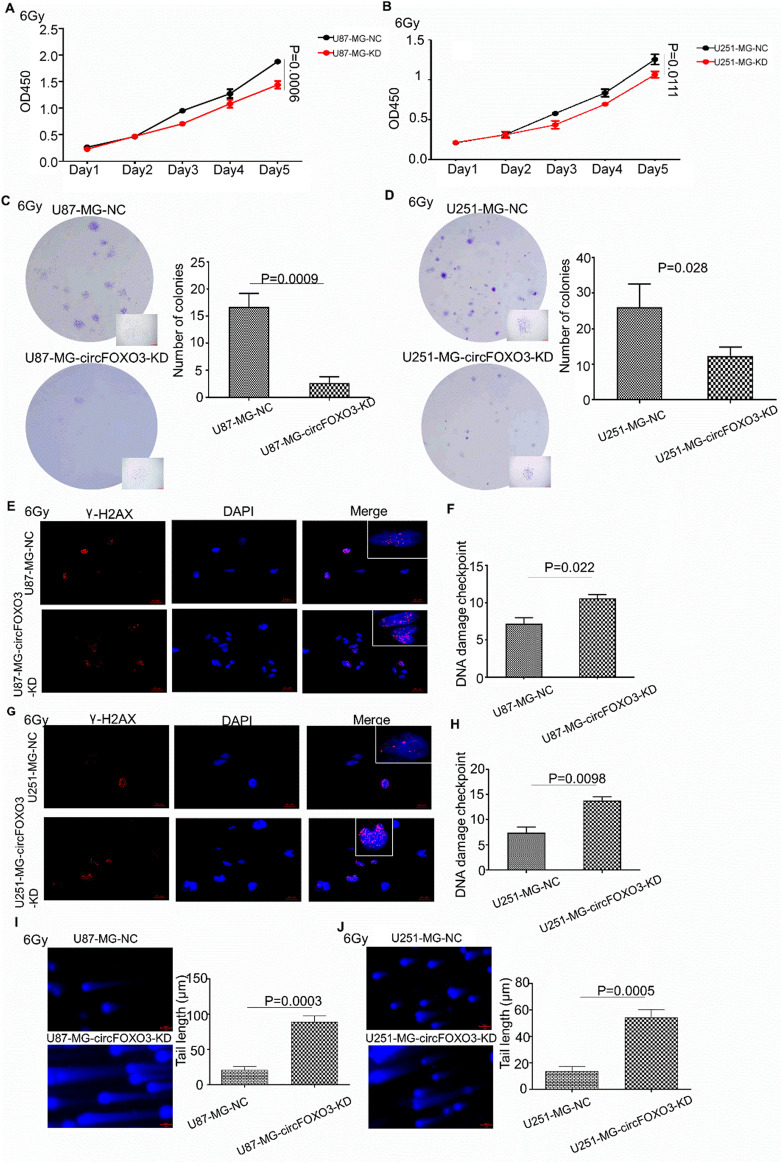
Knockdown of circFOXO3 in glioma cells suppressed proliferation and impaired DNA repair processes after irradiation. **(A, B)** Proliferation of U87-MG and U251-MG cells with or without circFOXO3 KD after 6 Gy irradiation. **(C, D)** Colony formation of U87-MG and U251-MG cells with or without circFOXO3 KD after 6 Gy irradiation. **(E–H)** γ-H2AX detection in circFOXO3-KD and NC U87-MG and U251-MG cells after 6 Gy irradiation. **(I, J)** Single-cell gel electrophoresis analysis of U87-MG and U251-MG cells with or without circFOXO3 KD after 6 Gy irradiation. The images in **(C–J)** represent three independent experiments. The data are presented as the mean ± SD with triplicate measurements for each group.

The impact of circFOXO3 on GBM cell proliferation and DNA damage repair was assessed with or without circFOXO3 KD. Cell Counting Kit-8 (CCK-8) assays revealed that inhibition of circFOXO3 significantly reduced cell proliferation under irradiation, particularly on the third to fifth days (Figures 2A, B). Colony formation assays further revealed that circFOXO3 KD significantly reduced the proliferative capacity of glioma cells under irradiation (Figures 2C, D). To evaluate DNA damage, single-cell electrophoresis and γ-H2AX immunofluorescence assays were performed to evaluate single- and double-strand DNA breaks *in vitro*. The “comet tail” length increased following irradiation (Figures 2E–H), indicating the induction of DNA strand breaks by X-ray. The percentage of tail DNA (%) was greater in circFOXO3-KD cells than in control cells, as shown in Figures 2E, G. Furthermore, γ-H2AX immunofluorescence analysis revealed an increased γ -H2AX signal at 24 h after irradiation in circFOXO3-KD cells, suggesting that circFOXO3 KD exacerbated radiation-induced DNA double-strand breaks (Figures 2I–J).

### 3.3 Overexpression of circFOXO3 promotes radioresistance in glioma cells

To further investigate whether circFOXO3 expression levels correlate with radioresistance, we overexpressed circFOXO3 in A172 and T98G cells, as shown in Figure 1F, and subsequently analyzed their potential radioresistance post- irradiation.

**FIGURE 3:**
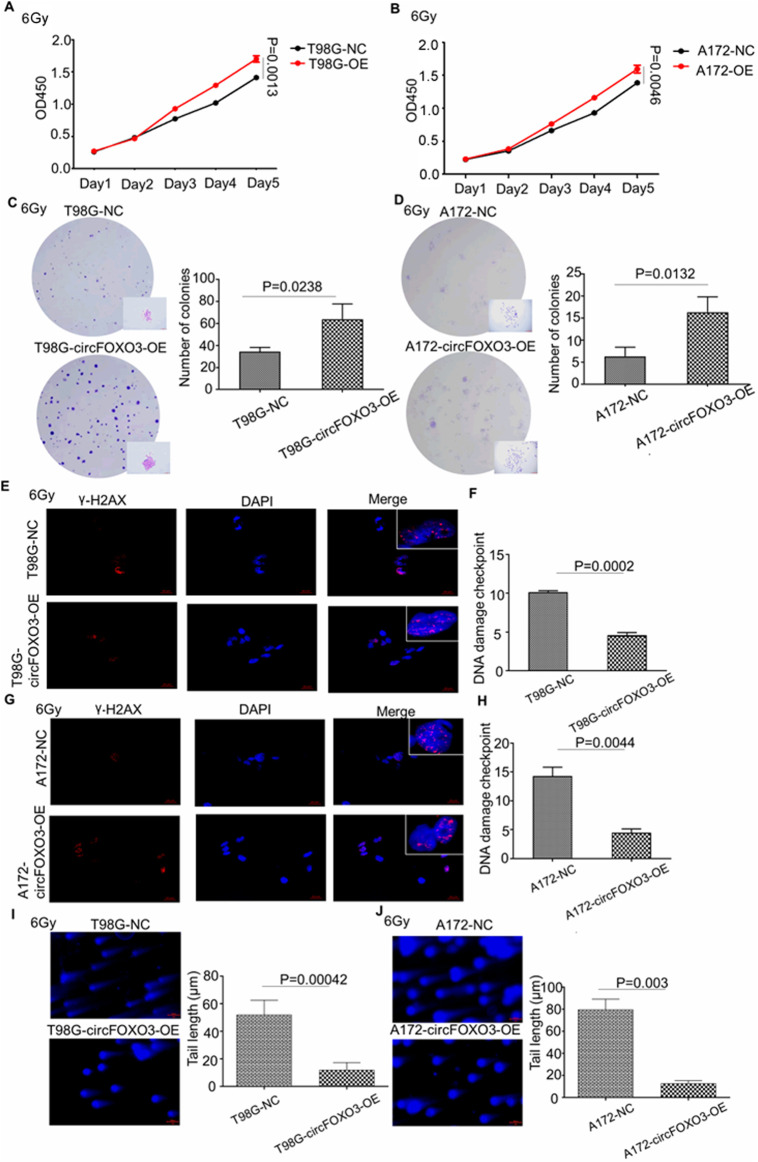
Overexpression of circFOXO3 increases the radioresistance of glioma cells *in vitro*. **(A, B)** Proliferation abilities of T98G and A172 cells with or without circFOXO3 OE after 6 Gy irradiation. **(C, D)** Colony formation of T98G and A172 cells with or without circFOXO3 OE after 6 Gy irradiation, **(E–H)** γ-H2AX detection in circFOXO3-OE and NC T98G and A172 cells after 6 Gy irradiation. **(I, J)** Single-cell gel electrophoresis analysis of T98G and A172 cells with or without circFOXO3 OE after 6 Gy irradiation. The images in **(C–J)** represent three independent experiments. The data are presented as the mean ± SD with triplicate measurements for each group.

Both the CCK-8 and colony formation assays demonstrated that high circFOXO3 expression significantly promoted cell proliferation after exposure to 6 Gy irradiation (Figures 3A–D). Additionally, single-cell electrophoresis and γ-H2AX immunofluorescence assays were performed on circFOXO3-OE cells. These assays revealed a reduction in “comet tail” length and decreased γ-H2AX signals following irradiation (Figures 3E–J), suggesting that circFOXO3 increases the DNA damage repair capacity of GBM cells post-irradiation.

### 3.4 Knockdown of circFOXO3 increases the radiosensitivity of GBM *in vivo*

**FIGURE 4:**
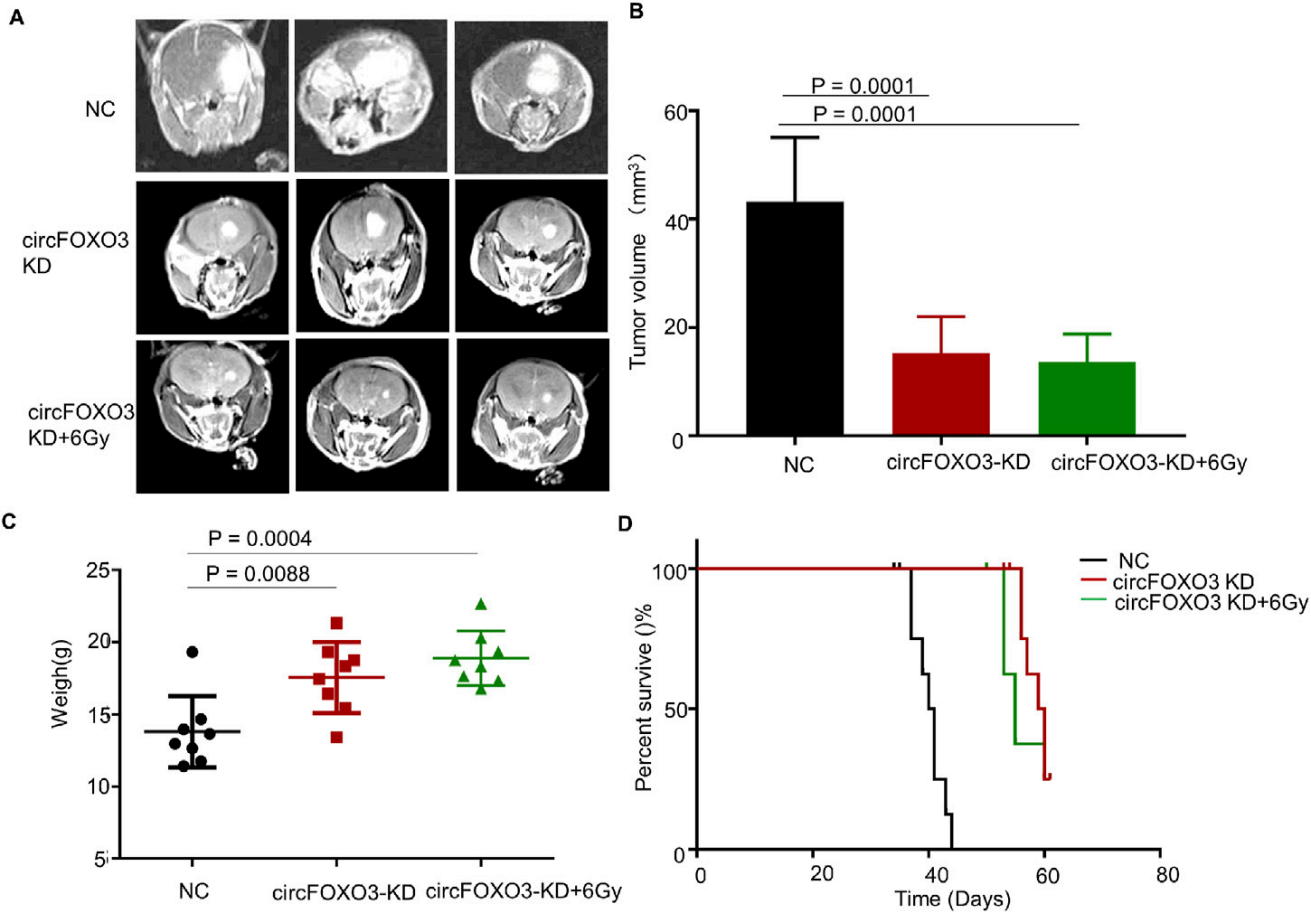
Knockdown of circFOXO3 increases radiosensitivity of GBM *in vivo*. **(A)** Representative MRI of xenograft GBM tumors orthotopically inoculated with U87-MG cells, with or without circFOXO3 KD, under irradiation (n = 8 per group). **(B)** Tumor volumes were calculated for each group using Coniglobus formula. **(C)** Weights of the mice in each group on the day of MRI detection. **(D)** Kaplan–Meier survival plots of the overall survival of the mice in the indicated groups (n = 8 per group). Survival curves were compared via Log-rank tests. Performed with Log-rank tests. All the data are presented as the mean ± SD.

To clarify the role of circFOXO3 *in vivo* and assess its impact on tumor resistance, we established a mouse xenograft model. When the nude mice developed neurological symptoms, the irradiation group was subjected to 6 Gy irradiation. MRI analysis revealed that, compared with the NC group, the circFOXO3 KD group exhibited significant inhibition of tumor growth and reduced tumor volume after irradiation (Figures 4A–C). Moreover, survival analysis revealed a remarkable extension in the lifespan of circFOXO3-KD mice, particularly following irradiation (Figure 4D).

These results indicated that circFOXO3 KD increased the radiosensitivity of GBM both *in vitro* and *in vivo*.

### 3.5 Metabolomic profiling of circFOXO3-overexpressing cells after irradiation

To evaluate the overall metabolic impact of radiotherapy on circFOXO3-OE cells, T98G-circFOXO3-OE cells were subjected to single doses of 6 Gy X-ray irradiation *in vitro*, followed by metabolomics profiling analysis. T98G cells were selected because of their sensitivity and stability in response to radiotherapy, which make them a suitable model for studying circFOXO3-mediated radioresistance.

**FIGURE 5:**
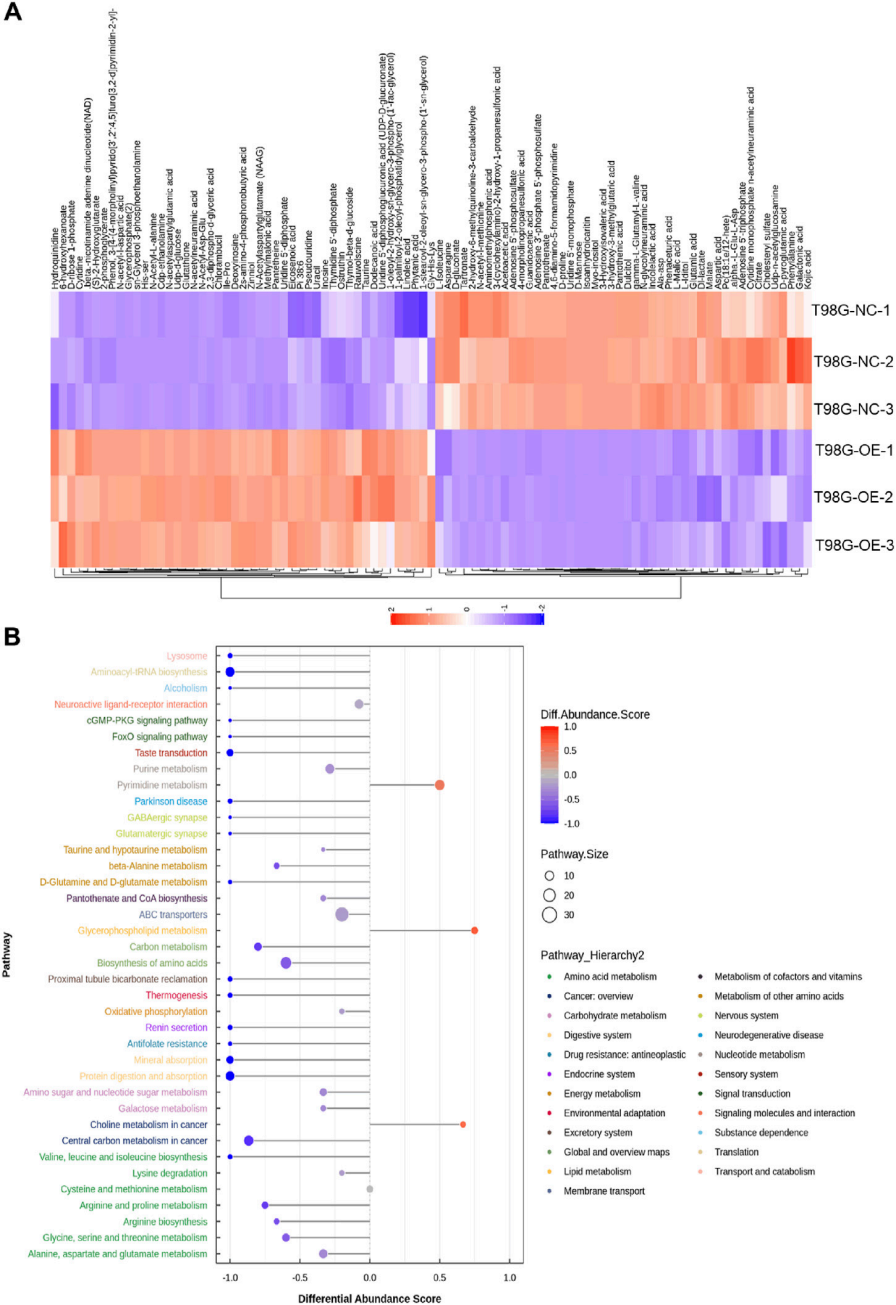
Metabolomic profiling of circFOXO3-overexpressing cells after irradiation. **(A)** Heatmap displaying differentially abundant metabolites. Each row represents a metabolite, and each column represents a sample. The color gradient reflects relative metabolite levels. **(B)** Hierarchical cluster heatmap of samples and differentially abundant metabolites.

Orthogonal partial least squares discriminant analysis plots were generated, as shown in Supplementary Figure S1A, B, which revealed that all samples in both positive and negative ion modes were closely clustered, indicating excellent experimental repeatability. Nontargeted LC–MS-based metabolomics identified 1,173 annotated metabolites, with 232 showing specific expression in T98G cells overexpressing circFOXO3 (Figure 5A). The volcano plot indicated that these identified metabolites exhibited significant differences in expression between T98G-circFOXO3-OE cells and the NC cells (VIP > 1 and p < 0.05) (Supplementary Figure S1C). Chemical classification revealed lipids and lipid-like molecules (26.854%), organic acids and derivatives (22.421%), and organoheterocyclic compounds (11.253%) as the three main categories of identified metabolites (Supplementary Figure S1D). Among the altered metabolites, glutathione, 6-hydroxyhexanoate, and chlorambucil were significantly upregulated in T98G-OE cells compared to control cells, whereas isoanhydroicaritin, 4,6-diamino-5-formamidopyrimidine, and pantothenate were notably downregulated.

**FIGURE 6:**
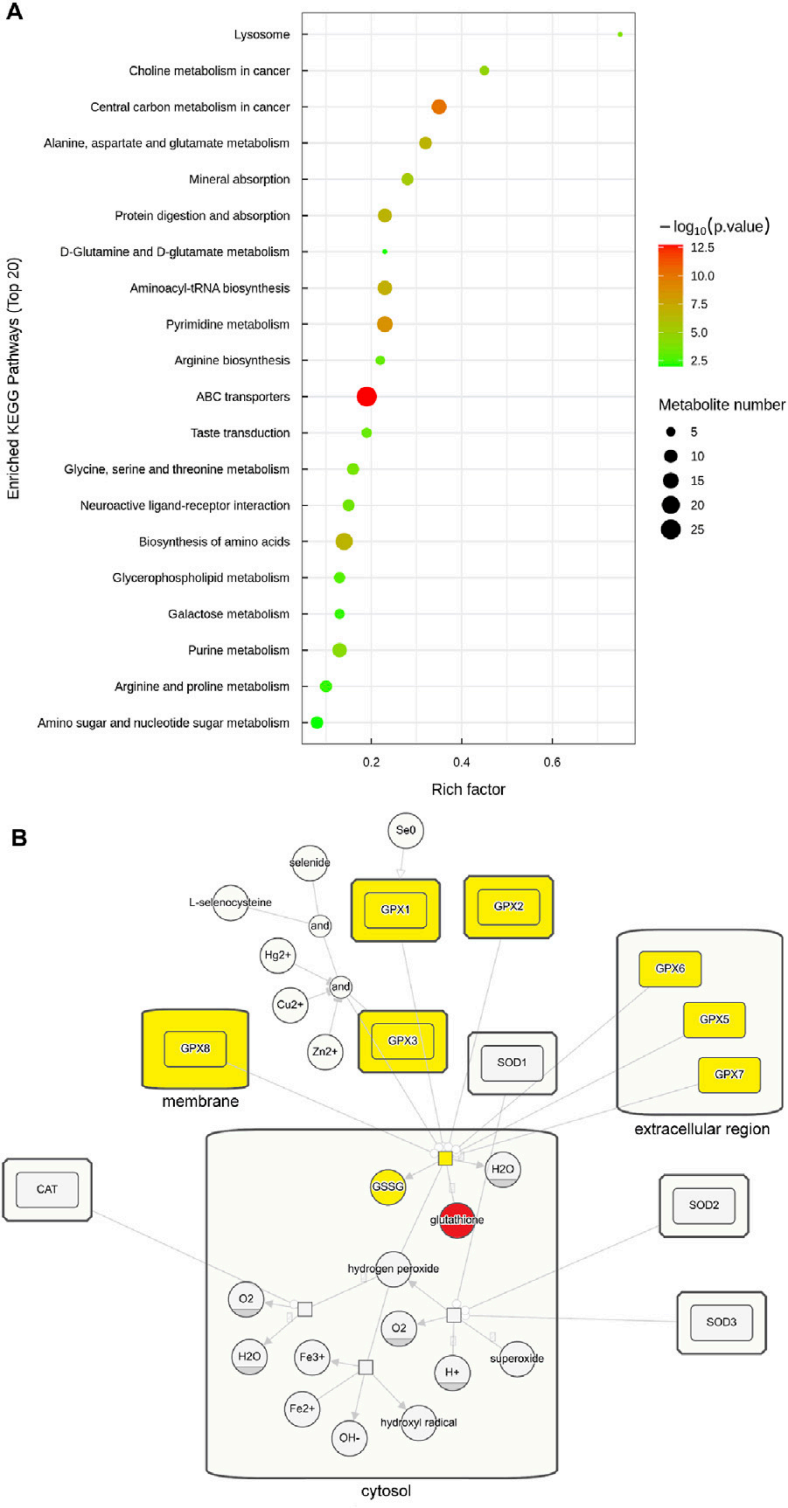
Enriched pathways of circFOXO3-overexpressing cells after irradiation. **(A)** Pathway enrichment analysis of significant differentially abundant metabolites. **(B)** Pathway map showing glutathione’s involvement in the ROS pathway. Glutathione is highlighted in red and related products and enzymes are highlighted in yellow to indicate their role.

The hierarchical cluster heatmap (Figure 5A) provided a visual representation of the differentially abundant metabolites across samples, while pathway enrichment analysis revealed that these metabolites were associated with pathways such as ABC transporters, central carbon metabolism in cancer, and pyrimidine metabolism (Figure 6A). The overall pathway changes were quantified using the differential abundance score (Figure 5B). Notably, the metabolite pathways enriched in the upregulated metabolites included pyrimidine metabolism, glycerophospholipid metabolism, and choline metabolism in cancer. Conversely, pathways such as ABC transporters, biosynthesis of amino acids, and purine metabolism were more enriched among the downregulated metabolites.

Among the significant alterations in metabolite profiles, elevated levels of glutathione were particularly notable. Glutathione is closely linked to ROS pathways in the cellular response to radiation. To further understand this connection, we examined the involvement of glutathione through KEGG pathway analysis, which is presented in Figure 6B.

### 3.6 Bioinformatics analysis of circFOXO3 and associated RNA-binding proteins

The secondary structure of circFOXO3 was predicted and is presented in Supplementary Figure S2B. CircMIR analysis revealed multiple microRNA (miRNA) binding sites within circFOXO3 (Supplementary Figure S2A). The primary miRNAs predicted to bind circFOXO3 were listed in Supplementary Table S1 and are visualized in Supplementary Figure S2A. Among these 59 miRNAs, eight were associated with glioma patient prognosis according to The Cancer Genome Atlas (TCGA) database analysis. Additionally, circFOXO3 was found to interact with 8 RNA-binding proteins (RBPs), as identified by circAtlas and CircRNA Interactome (Supplementary Figure S2C). These RBPs were enriched predominantly in biological processes such as positive regulation of translation, RNA splicing, and mRNA processing. With respect to molecular function, the RBPs were associated primarily with RNA binding, mRNA binding, and DNA binding (Supplementary Figure S2D). These results revealed that circFOXO3 exerts its functional effects through interactions with both miRNAs and RBPs.

**FIGURE 7:**
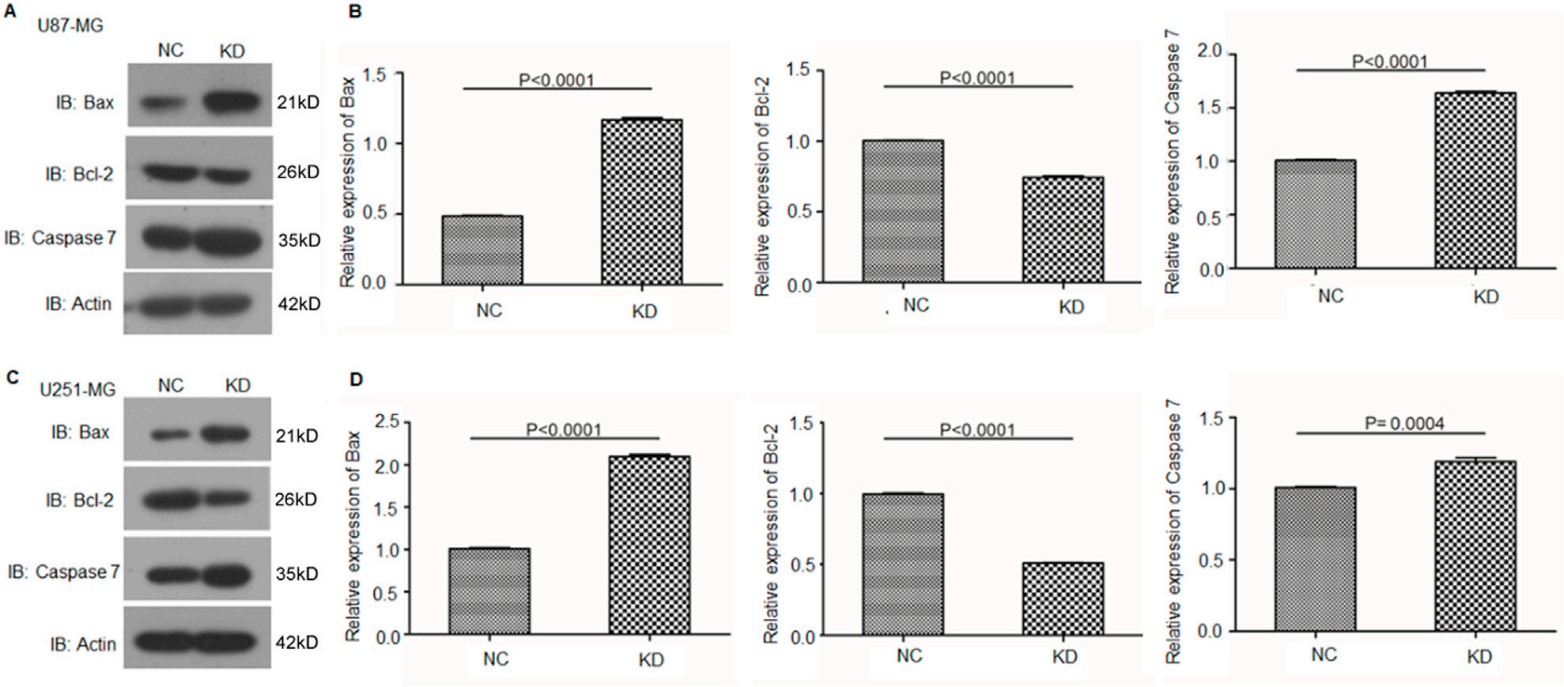
Knockdown of circFOXO3 increases the expression of apoptotic proteins after irradiation. **(A, B)** Western blot analysis of Bax, Bcl-2, and caspase-7 expression in U87-MG cells with or without circFOXO3 KD. **(C, D)** Similar analysis in U251-MG cells. The data are presented as the mean ± SD.

Given that oxidative stress can regulate the apoptosis of glioma cells via the Caspase pathway (Wen et al., 2023), we further investigated the expression levels of apoptosis-related proteins, including caspase-7, Bcl-2, and Bax, in circFOXO3-KD and NC cells. Western blot analysis revealed a significant increase in the expression of caspase-7 and Bax, along with a notable decrease in the Bcl-2 level (Figure 7). These findings indicate that the suppression of circFOXO3 promotes apoptosis-related signaling in glioma cells following 6 Gy irradiation.

## 4 Discussion

GBM is considered one of the most prevalent primary tumors in the central nervous system. Recent studies have suggested that circRNAs play a significant role in tumor radioresistance by regulating key processes such as DNA damage repair, apoptosis, and cell cycle changes under hypoxic conditions (Lei et al., 2019; Liu et al., 2019; Zhao et al., 2019; Wei et al., 2019; Yang et al., 2019; Yu et al., 2018). In light of these findings, we conducted a series of experiments to assess the function of circFOXO3 in GBM radiosensitivity and performed metabolomics profiling to reveal the underlying mechanism.

Our metabolomics profiling revealed significant alterations in several key metabolites and metabolic pathways, which may contribute to the observed radioresistance in GBM cells. Notably, the elevated levels of glutathione, a critical antioxidant, is closely linked to the ROS pathway in the cellular response to irradiation. ROS are produced as a result of direct high-energy ionizing radiation and indirect radiation hydrolysis. These highly reactive molecules induce oxidative stress, causing damage to cellular DNA, membranes, and proteins, ultimately leading to cell death (Zheng et al., 2023; Luo et al., 2018). Additionally, radiotherapy can promote lipid peroxidation by producing a large number of ROS, upregulating the expression of key enzyme ACSL4, which in turn can lead to ferroptosis (Phadnis et al., 2023). Additionally, the enrichment of metabolites in pathways such as pyrimidine metabolism, glycerophospholipid metabolism, choline metabolism, purine metabolism, and galactose metabolism highlights the extensive metabolic reprogramming in GBM cells. Tumor cells extensively adopt distinct metabolic processes, including the rewired glucose, amino acid, and lipid metabolism, to meet their demands for rapid growth and survival. This metabolic flexibility contributes to tumor progression and may also underlie the development of radioresistance. Indeed, recent studies have shown that glycolysis-to-fatty acid oxidation metabolic rewiring is associated with radioresistant GBM cells and regrown tumors after radiation in syngeneic mice (Jiang et al., 2022). Additionally, radiation-induced rewiring of glucose metabolism has been implicated in altering the radiosensitivity of GBM cells (Bailleul et al., 2023). In addition, our metabolomics analysis revealed that lipids and lipid-like molecules (26.854%), organic acids and derivatives (22.421%), and organoheterocyclic compounds (11.253%) were the three predominant classes of metabolites. These findings suggest that targeting specific metabolic plasticity regulators or pathways could be a promising strategy to enhance the efficacy of radiotherapy in glioma patients.

Although our study provides valuable insights into the potential mechanisms of circFOXO3-mediated radioresistance through bioinformatics analysis of circFOXO3 binding miRNAs and proteins, as well as metabolomics profiling in circFOXO3-overexpressing cells, further research is needed to elucidate the specific downstream targets or pathways affected by circFOXO3. Additionally, advanced delivery systems such as nanoparticle-based therapies, which can achieve selectively delivery, could be explored to accelerate clinical transformation.

**FIGURE 8:**
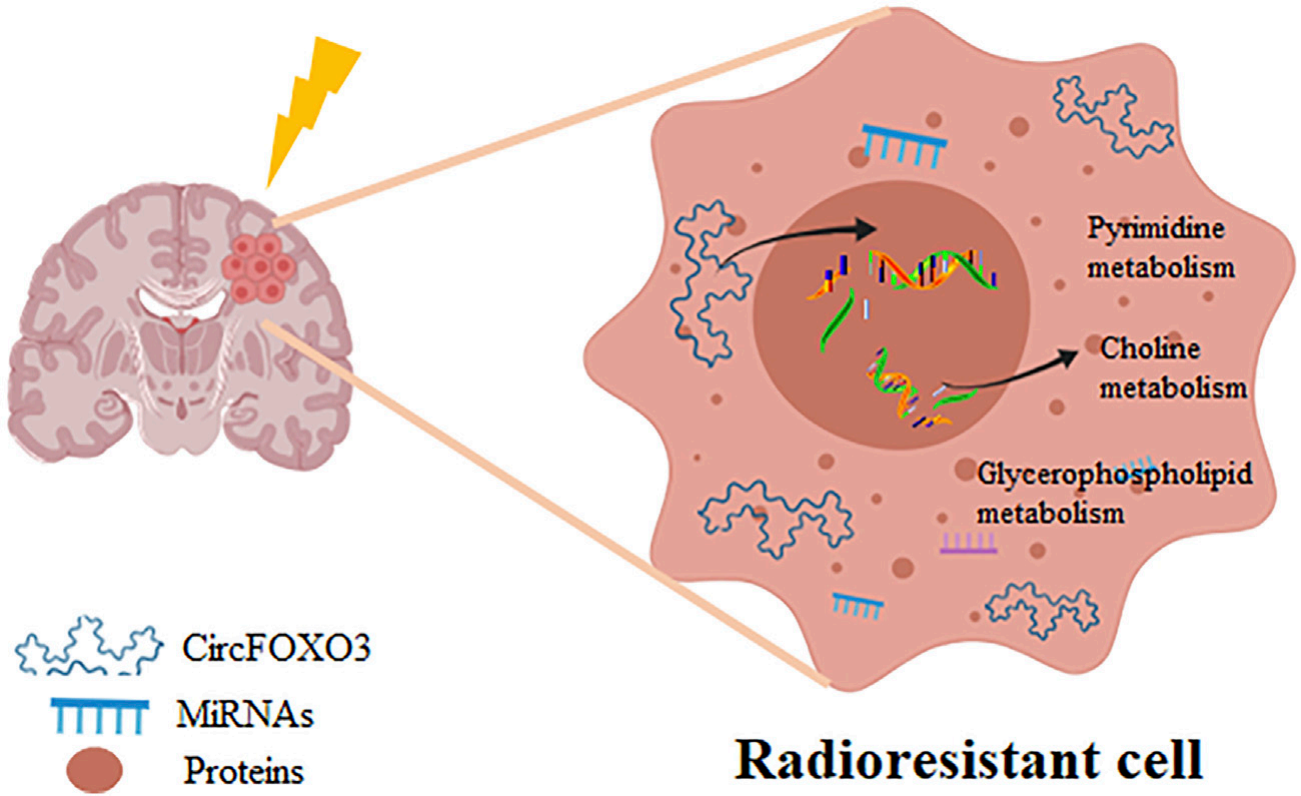
CircFOXO3 regulates multiple metabolic pathways involved in glioblastoma radioresistance.

In conclusion, our study is the first to establish that the upregulation of circFOXO3 promotes radioresistance in glioma by modulating the levels of key metabolites and the activity of metabolic pathways (Figure 8). These findings suggest that circFOXO3 may serve as a potential diagnostic and therapeutic target in gliomas.

## Data Availability

The original contributions presented in the study are included in the article/Supplementary Material, further inquiries can be directed to the corresponding authors.

## References

[B1] Bailleul J. Ruan Y. Abdulrahman L. Scott A. J. Yazal T. Sung D. (2023). M2 isoform of pyruvate kinase rewires glucose metabolism during radiation therapy to promote an antioxidant response and glioblastoma radioresistance. Neuro Oncol. 25 (11), 1989–2000. 10.1093/neuonc/noad103 37279645 PMC10628945

[B2] Gupta K. Vuckovic I. Zhang S. Xiong Y. Carlson B. L. Jacobs J. (2020). Radiation induced metabolic alterations associate with tumor aggressiveness and poor outcome in glioblastoma. Front. Oncol. 10, 535. 10.3389/fonc.2020.00535 32432031 PMC7214818

[B3] Jiang N. Xie B. Xiao W. Fan M. Xu S. Duan Y. (2022). Fatty acid oxidation fuels glioblastoma radioresistance with CD47-mediated immune evasion. Nat. Commun. 13 (1), 1511. 10.1038/s41467-022-29137-3 35314680 PMC8938495

[B4] Kang H. Kim B. Park J. Youn H. Youn B. (2023). The Warburg effect on radioresistance: survival beyond growth. Biochim. Biophys. Acta Rev. Cancer 1878 (6), 188988. 10.1016/j.bbcan.2023.188988 37726064

[B5] Lei F. Lei T. Huang Y. Yang M. Liao M. Huang W. (2019). Radio-Susceptibility of nasopharyngeal carcinoma: focus on epstein-barr virus, MicroRNAs, long non-coding RNAs and circular RNAs. Curr. Mol. Pharmacol. 13, 192–205. 10.2174/1874467213666191227104646 31880267

[B6] Liao H. Yang S. Liang Z. Xiao L. Xie S. Lin P. (2023). A cancer cell selective replication stress nano amplifier promotes replication fork catastrophe to overcome radioresistance. ACS Nano 17 (18), 18548–18561. 10.1021/acsnano.3c06774 37706454

[B7] Liu J. Xue N. Guo Y. Niu K. Gao L. Zhang S. (2019). CircRNA_100367 regulated the radiation sensitivity of esophageal squamous cell carcinomas through miR-217/Wnt3 pathway. Aging (Albany NY) 11, 12412–12427. 10.18632/aging.102580 31851619 PMC6949088

[B8] Liu S. Wang W. Hu S. Jia B. Tuo B. Sun H. (2023). Radiotherapy remodels the tumor microenvironment for enhancing immunotherapeutic sensitivity. Cell Death Dis. 14 (10), 679. 10.1038/s41419-023-06211-2 37833255 PMC10575861

[B9] Lu W. Y. (2017). Roles of the circular RNA circ-Foxo3 in breast cancer progression. Cell Cycle 16, 589–590. 10.1080/15384101.2017.1278935 28278047 PMC5397257

[B10] Luo M. Shang L. Brooks M. D. Jiagge E. Zhu Y. Buschhaus J. M. (2018). Targeting breast cancer stem cell state equilibrium through modulation of redox signaling. Cell Metab. 28 (1), 69–86. 10.1016/j.cmet.2018.06.006 29972798 PMC6037414

[B11] Ostrom Q. T. Bauchet L. Davis F. G. Deltour I. Fisher J. L. Langer C. E. (2014). The epidemiology of glioma in adults: a “state of the science” review. Neuro-oncology 16, 896–913. 10.1093/neuonc/nou087 24842956 PMC4057143

[B12] Ostrom Q. T. Price M. Neff C. Cioffi G. Waite K. A. Kruchko C. (2022). CBTRUS statistical report: primary brain and other central nervous system tumors diagnosed in the United States in 2015–2019. Neuro-oncology 24, v1–v95. 10.1093/neuonc/noac202 36196752 PMC9533228

[B13] Phadnis V. V. Snider J. Varadharajan V. Ramachandiran I. Deik A. A. Lai Z. W. (2023). MMD collaborates with ACSL4 and MBOAT7 to promote polyunsaturated phosphatidylinositol remodeling and susceptibility to ferroptosis. Cell Rep. 42 (9), 113023. 10.1016/j.celrep.2023.113023 37691145 PMC10591818

[B14] Rodriguez-Colman M. J. Dansen T. B. Burgering B. M. T. (2024). FOXO transcription factors as mediators of stress adaptation. Nat. Rev. Mol. Cell Biol. 25 (1), 46–64. 10.1038/s41580-023-00649-0 37710009

[B15] Sedlackova L. Kataura T. Sarkar S. Korolchuk V. I. (2023). Metabolic function of autophagy is essential for cell survival. Autophagy 19 (8), 2395–2397. 10.1080/15548627.2023.2165753 36727253 PMC10351444

[B16] Su Y. Zhu C. Wang B. Zheng H. McAlister V. Lacefield J. C. (2021). Circular RNA Foxo3 in cardiac ischemia-reperfusion injury in heart transplantation: a new regulator and target. Am. J. Transpl. 21 (9), 2992–3004. 10.1111/ajt.16475 33382168

[B17] van den Bent M. J. Geurts M. French P. J. Smits M. Capper D. Bromberg J. E. C. (2023). Primary brain tumours in adults. Lancet (23), S0140–S6736. 10.1016/S0140-6736(23)01054-1 37738997

[B18] Wei L. Wang X. Lv L. Liu J. Xing H. Song Y. (2019). The emerging role of microRNAs and long noncoding RNAs in drug resistance of hepatocellular carcinoma. Mol. Cancer 18, 147. 10.1186/s12943-019-1086-z 31651347 PMC6814027

[B19] Wen Z. H. Kuo H. M. Shih P. C. Hsu L. C. Chuang J. M. Chen N. F. (2023). Isoaaptamine increases ROS levels causing autophagy and mitochondria-mediated apoptosis in glioblastoma multiforme cells. Biomed. Pharmacother. 160, 114359. 10.1016/j.biopha.2023.114359 36753955

[B20] Yang W. Liu Y. Gao R. Xiu Z. Sun T. (2019). Knockdown of cZNF292 suppressed hypoxic human hepatoma SMMC7721 cell proliferation, vasculogenic mimicry, and radioresistance. Cell Signal 60, 122–135. 10.1016/j.cellsig.2019.04.011 31028816

[B21] Yu D. Li Y. Ming Z. Wang H. Dong Z. Qiu L. (2018). Comprehensive circular RNA expression profile in radiation-treated HeLa cells and analysis of radioresistance-related circRNAs. PeerJ 6, e5011. 10.7717/peerj.5011 29922514 PMC6005163

[B22] Zhang Y. Zhao H. Zhang L. (2018). Identification of the tumorsuppressive function of circular RNA FOXO3 in nonsmall cell lung cancer through sponging miR155. Mol. Med. Rep. 17, 7692–7700. 10.3892/mmr.2018.8830 29620202 PMC5983971

[B23] Zhao M. Xu J. Zhong S. Liu Y. Xiao H. Geng L. (2019). Expression profiles and potential functions of circular RNAs in extracellular vesicles isolated from radioresistant glioma cells. Oncol. Rep. 41, 1893–1900. 10.3892/or.2019.6972 30664179

[B24] Zheng Z. Su J. Bao X. Wang H. Bian C. Zhao Q. (2023). Mechanisms and applications of radiation-induced oxidative stress in regulating cancer immunotherapy. Front. Immunol. 14, 1247268. 10.3389/fimmu.2023.1247268 37600785 PMC10436604

