# Supplementary material for: CircFOXO3 upregulation mediates the radioresistance of glioblastoma by affecting cellular metabolome
Source: Front Pharmacol. 2024 Oct 10;15:1479480. doi: 10.3389/fphar.2024.1479480 (PMC11499195; doi:10.3389/fphar.2024.1479480)
Supplement: Supplementary file 2 [file DataSheet1.DOCX]

**Supplementary Materials**

for

**CircFOXO3 Upregulation Mediates the Radioresistance of Glioblastoma by Affecting Cellular Metabolome**

Hao Xu^1, #^, Jin Xing^1, #^, Lilin Cheng^2, #^, Zhihan Wang^1^, Liang Zhao^1^, Li Ren^1,^ *, Shuai Zhang^2,^ *

1. Department of Neurosurgery, Shanghai Pudong Hospital, Fudan University Pudong Medical Center, 2800 Gongwei Road, Pudong, Shanghai 201399, China;

2. Department of Neurosurgery, Changhai Hospital, Naval Medical University, NO.168 Changhai Road, Shanghai, 200433, China.

#These authors contributed equally to this work.

**Co-corresponding authors**

Shuai Zhang, Department of Neurosurgery, Changhai Hospital, Naval Medical University, NO.168 Changhai Road, Shanghai, 200433, China. Phone:186-158-61672371, E-mail: zhang_dream@yeah.net.

Renli, Department of Neurosurgery, Shanghai Pudong Hospital, Fudan University Pudong Medical Center, 2800 Gongwei Road, Pudong, Shanghai 201399, China; Phone:186-136-36499356, E-mail: [slysw@sina.com](mailto:slysw@sina.com).

**Supplementary Materials and Methods**

**1. Cell Culture**

Glioma cell lines (U251-MG, U87-MG, T98G, and A172) were obtained from the Cell Bank of the Shanghai Branch of the Chinese Academy of Sciences. Cells were cultured in Dulbecco’s Modified Eagle Medium (DMEM) supplemented with 10% fetal bovine serum (FBS, Invitrogen) and maintained at 37°C in a humidified atmosphere containing 5% CO2. Cells were regularly monitored and maintained in a logarithmic growth phase for experimental use.

**2. Quantitative Real-Time RT-PCR (qRT-PCR)**

Total RNA was extracted using Trizol reagent (Invitrogen). Then qRT-PCR was performed as follows: an equal amount of total RNA was used for cDNA synthesis using the oligo-dT primer and M-myeloblastosis virus reverse transcriptase XL (Promega). The synthesized cDNA (2 μl) was used for each PCR. The RT-PCR primers for circFOXO3 were: F: 5'-attgtccatggagacagcccgccg-3' R, 5'-gtggggaacttcactggtgctaag-3'.

**3. Western Blot Analysis**

The proteins (30 μg) were subjected to 8-10 % gel electrophoresis and transferred onto Hybond ECL membranes (Amersham). The membranes were incubated for 1 h at room temperature in blocking buffer (5 % skim milk in TBS-T) and then incubated with following antibodies (dilution1:1000; anti-Bcl-2 antibody (cat 15071, Cell Signalling Technology, USA); anti-caspase-7 antibody (cat 9492, Cell Signalling Technology, USA); anti-Bax antibody (cat 2772, Cell Signalling Technology, USA); anti- β-actin antibody) overnight at 4°C. After washing with TBS-T, the membranes were incubated with horseradishperoxidase-conjugated anti-rabbit or anti-mouse antibody (1:10,000 dilution; Sigma) for 2 h at RT. Detection was performed using Western blot detection reagents (Odyssey).

**4. Detailed Untargeted Metabolomics Analysis**

To investigate the effect of circFOXO3 overexpression after irradiation on glioma cells metabolites, 6 samples of T98G-circFOXO3-OE and NC cells were taken for untargeted metabolomics analysis. Briefly, samples were extracted for analysis and re-dissolved in 100 μL acetonitrile/water (1:1, v/v) solvent for LC-MS Analysis, extracts were analyzed using a quadrupole time-of-flight mass spectrometer (Sciex TripleTOF 6600) coupled to hydrophilic interaction chromatography via electrospray ionization in Shanghai Applied Protein Technology Co., Ltd. As for Data Analysis,the raw MS data (wiff.scan files) were converted to MzXML files using ProteoWizard MSConvert before importing into freely available XCMS software. VIP value >1 and p<0.05 was considered as statistically significant.

The metabolites were blasted against the online KEGG database to retrieve their COs and were subsequently mapped to pathways in KEGG. For hierarchical clustering, Cluster 3.0 (http://bonsai.hgc.jp/~mdehoon/software/cluster/software.htm) and the Java Treeview software (http://jtreeview.sourceforge.net) were used.

**5. Lentiviral vector-mediated gene knockdown and overexpression**

To construct circFOXO3 OE plasmids, a basic sequence (flanked by HxoI and Agel) was synthesized. A small spacer sequence containing two restriction enzyme sites, HindIII and SalI, was added for the insertion of the circRNA fragment. The circFOXO3 KD target sequences were 5’GGGCAAAGCAGAACUCCAUUU3’. The recombinant lentivirus and the NC lentivirus (Hanyin Co. Shanghai, China) were prepared and titered to 10^9^ TU/ml (transfection unit). U87-MG and A172 cells were infected with the same titre virus with 8 μg/ml polybrene. Approximately 72 h later, the culture medium was replaced with selection medium containing 4 μg/ml puromycin. The cells were then cultured for at least 14 days. The puromycin-resistant cells were amplified in medium containing 2 μg/ml puromycin for seven to nine days and then transferred to a medium without puromycin. The KD or OE efficiency was evaluated by qRT-PCR.

**6. Bioinformatic Analysis**

CircMIR (https://www.bio-inf.cn/circmir/) was used to predict circFOXO3-miRNA interactions based on the RNAhybrid (https://bibiserv.cebitec.uni-bielefeld.de/rnahybrid/) and TargetScan (https://www.targetscan.org/) databases. The circFOXO3-protein interactions were predicted based on circAtlas (https://circatlas.biols.ac.cn/) and Circular RNA Interactome (https://circinteractome.irp.nia.nih.gov/).


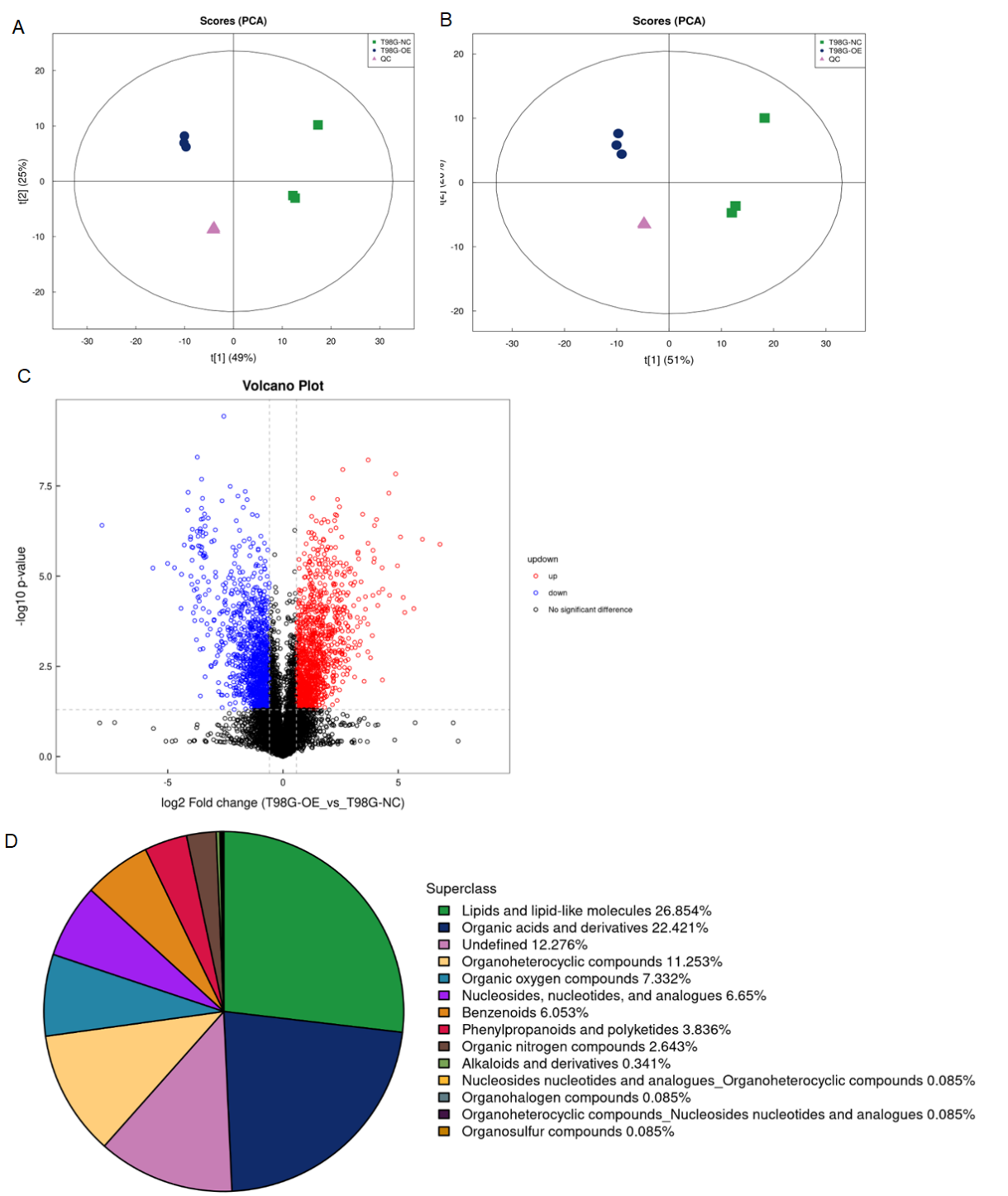
 **Figure S1. Metabolic shifts in glioma cells with or without irradiation.**

**A, B** Score plots from supervised orthogonal partial least squares discriminant analysis of metabolomic data. **C** Volcano plot showing the log2 fold change (FC) versus the -Log10 p-value for metabolites (VIP > 1). **D** Pie chart representing the subclass distribution of differentially expressed metabolites (VIP > 1 and p-value < 0.05).


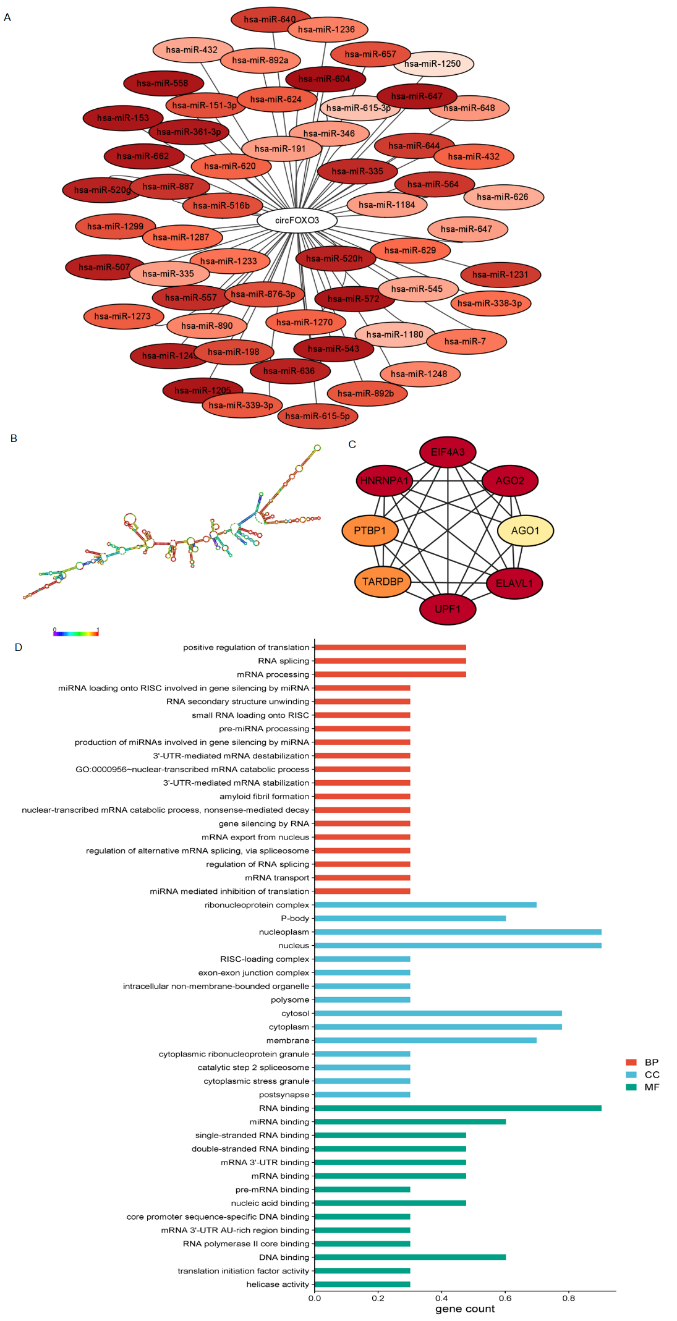


**Figure S2. Functional enrichment analysis of circFOXO3 interactions.**

**A** Circular network of circFOXO3-miRNA interactions. **B** Predicted secondary structure of circFOXO3. **C** Network of CircFOXO3 interaction with 8 RBPs by circAtlas and CircRNA Interactome. **D** GO enrichment analysis of RBPs associated with circFOXO3, categorized into biological process (BP), cellular component (CC), and molecular function (MF).

**Table S1. Predicted miRNAs that bind to circFOXO3.**
